# Integration of genomic copy number variations and chemotherapy-response biomarkers in pediatric sarcoma

**DOI:** 10.1186/s12920-018-0456-5

**Published:** 2019-01-31

**Authors:** Lijun Cheng, Pankita H. Pandya, Enze Liu, Pooja Chandra, Limei Wang, Mary E. Murray, Jacquelyn Carter, Michael Ferguson, Mohammad Reza Saadatzadeh, Khadijeh Bijangi-Visheshsaraei, Mark Marshall, Lang Li, Karen E. Pollok, Jamie L. Renbarger

**Affiliations:** 1 Department of Biomedical Informatics, College of Medicine, Ohio State University, Columbus, OH 43210 USA; 2 Herman B Wells Center for Pediatric Research, Department of Pediatrics, School of Medicine, Indiana University, Indianapolis, IN 46202 USA; 3 Division of Hematology/Oncology, Department of Pediatrics, School of Medicine, Indiana University, Indianapolis, IN 46202 USA; 4 Center for Computational Biology and Bioinformatics, School of Medicine, Indiana University, Indianapolis, IN 46202 USA; 5 Indiana University Melvin and Bren Simon Cancer Center, Indianapolis, IN 46202 USA; 6 Indiana Institute of Personalized Medicine, Indiana University, Indianapolis, IN 46202 USA

**Keywords:** Copy number variation, Pediatric sarcomas, Precision medicine, Prognostic biomarkers, Comparative genomic hybridization-array

## Abstract

**Background:**

While most pediatric sarcomas respond to front-line therapy, some bone sarcomas do not show radiographic response like soft-tissue sarcomas (rhabdomyosarccomas) but do show 90% necrosis. Though, new therapies are urgently needed to improve survival and quality of life in pediatric patients with sarcomas. Complex chromosomal aberrations such as amplifications and deletions of DNA sequences are frequently observed in pediatric sarcomas. Evaluation of copy number variations (CNVs) associated with pediatric sarcoma patients at the time of diagnosis or following therapy offers an opportunity to assess dysregulated molecular targets and signaling pathways that may drive sarcoma development, progression, or relapse. The objective of this study was to utilize publicly available data sets to identify potential predictive biomarkers of chemotherapeutic response in pediatric Osteosarcoma (OS), Rhabdomyosarcoma (RMS) and Ewing’s Sarcoma Family of Tumors (ESFTs) based on CNVs following chemotherapy (OS *n* = 117, RMS *n* = 64, ESFTs *n* = 25 tumor biopsies).

**Methods:**

There were 206 CNV profiles derived from pediatric sarcoma biopsies collected from the public databases TARGET and NCBI-Gene Expression Omnibus (GEO). Through our comparative genomic analyses of OS, RMS, and ESFTs and 22,255 healthy individuals called from the Database of Genomic Variants (DGV), we identified CNVs (amplifications and deletions) pattern of genomic instability in these pediatric sarcomas. By integrating CNVs of Cancer Cell Line Encyclopedia (CCLE) identified in the pool of genes with drug-response data from sarcoma cell lines (*n* = 27) from Cancer Therapeutics Response Portal (CTRP) Version 2, potential predictive biomarkers of therapeutic response were identified.

**Results:**

Genes associated with survival and/recurrence of these sarcomas with statistical significance were found on long arm of chromosome 8 and smaller aberrations were also identified at chromosomes 1q, 12q and x in OS, RMS, and ESFTs. A pool of 63 genes that harbored amplifications and/or deletions were frequently associated with recurrence across OS, RMS, and ESFTs. Correlation analysis of CNVs from CCLE with drug-response data of CTRP in 27 sarcoma cell lines, 33 CNVs out of 63 genes correlated with either sensitivity or resistance to 17 chemotherapies from which actionable CNV signatures such as IGF1R, MYC, MAPK1, ATF1, and MDM2 were identified. These CNV signatures could potentially be used to delineate patient populations that will respond versus those that will not respond to a particular chemotherapy.

**Conclusions:**

The large-scale analyses of CNV-drug screening provides a platform to evaluate genetic alterations across aggressive pediatric sarcomas. Additionally, this study provides novel insights into the potential utilization of CNVs as not only prognostic but also as predictive biomarkers of therapeutic response. Information obtained in this study may help guide and prioritize patient-specific therapeutic options in pediatric bone and soft-tissue sarcomas.

**Electronic supplementary material:**

The online version of this article (10.1186/s12920-018-0456-5) contains supplementary material, which is available to authorized users.

## Background

Sarcomas are a rare form of soft-tissue and/or bone cancers [1]. Osteosarcoma (OS), Rhabdomyosarcoma (RMS) and Ewing sarcoma family of tumors (ESFTs) are the three most common types of sarcomas that affect mostly children and teenagers, and account for approximately 15% of all childhood malignancies in the United States [2–6]. Only 30% of relapsed/recurrent OS, RMS and ESFTs patients benefit from neoadjuvant chemotherapy [7–9]. Thus, it is imperative to identify predictive biomarkers of chemotherapeutic response in these pediatric sarcoma patients to improve prognosis and clinical outcomes. This will ultimately help to stratify patient populations that will respond to chemotherapy based on their molecular landscape.

Genetic variation is one of many characteristics of pediatric sarcomas [10–13]. It has been reported that DNA copy number variations (CNVs) and gene fusions lead to altered gene expression and eventually contribute to the development of sarcoma [10–13]. There are 55 DNA structure variation sets listed as standard clinical diagnostic biomarkers for sarcoma by the medical leader report of National Comprehensive Cancer Network (NCCN) Biomarkers Compendium [https://www.nccn.org/professionals/biomarkers/default.aspx]. Forty-five out of 55 are fusion gene variations, while 3 of the 55 genetic alterations are CNVs. However, none of these alterations have been approved as predictive biomarkers for first line chemotherapy treatment in pediatric sarcomas [https://www.nccn.org/professionals/biomarkers/default.aspx] (Additional file 1). Many of these chromosomal changes may be responsible for pediatric sarcoma progression and relapse, but the underlying cause of the large number of copy number amplifications and deletions remains unclear. Genetic alterations that serve as prognostic biomarkers for aggressive pediatric sarcomas will be investigated to also determine if they can be used as predictive biomarkers of therapeutic response.

OS is the most common primary malignant bone tumor in children and adolescents and is characterized by complex deregulated signaling [5, 7]. Comprehensive molecular profiling of OS shows copy number amplification and overexpression of genes in chr8 and chr17p11.2-p12 that strongly correlate with OS progression and relapse [5, 7]. Amplification of MET, CCNE1, and PDGFRα genes provides promising prognostic biomarkers for tailoring personalized therapies for OS patients [14]. Notably, ESFT is the second most common primary malignant bone tumor in children and adolescents. The most frequent copy number gains are observed in whole chr 8 and chr 12, long arm of chr 1. Copy number loss is commonly observed on the long arm (q) of chr 16 correlates with shorter survival in ESFTs [15, 16]. RMS is the most common soft tissue sarcoma in children. The frequent gains and amplifications associated with short-term survival include 12q13.3-q14.1 and 8p11.1–11.2 which harbor CDK4, MYCN, GLI, MDM2, FGFR1, and FGFR4 genes [17, 18]. Most of these biomarkers have been proposed for a specific sarcoma subtypes (OS, ESFTs, RMS) but not for all three pediatric sarcomas. These prognostic biomarkers still need to be evaluated via genome-wide studies for their role as potential predictive biomarkers of therapeutic responses across multiple pediatric sarcoma subtypes.

There is still a critical need for elucidating predictive biomarkers of therapeutic response for progressive pediatric sarcomas. To this end, prognostic biomarkers of pediatric sarcomas have the potential to also serve as predictive biomarkers of therapeutic responses, which would help guide and prioritize patient-specific therapeutic options. As mentioned above, chromosomal aberrations such as DNA copy number amplifications and deletions are frequently observed in pediatric sarcomas and can be retrospectively integrated with drug response data to ultimately allow for predictions of response to chemotherapies. For our study, we focused our efforts on exploiting high-resolution array Comparative Genomic Hybridization (aCGH) [19] to distinguish such pediatric sarcoma-associated CNVs pattern in OS, RMS, and ESFTs. This included comparison of chromosome bands and genes in pediatric sarcomas to, healthy population CNVs in the Database of Genomic Variants (DGV) [20]. Comprehensive literature reviews were also conducted to collect CNV amplifications and deletions of many genes from the PubMed repository which may serve as a tool for predicting clinical outcomes in all three types of sarcomas.

Genomic variations can contribute to differences in cancer cell drug responses. Systematic cell line-based platforms provide an important resource to evaluate the therapeutic efficacy of candidate anticancer agents for sarcomas harboring similar genetic alterations such as chromosomal CNVs. In the present study, we conducted a comprehensive CNV profile comparison between sarcoma cell lines and patient tumors. CNVs in 63 genes that serve as prognostic biomarkers of pediatric sarcomas were evaluated to determine if they correlated with sensitivity or resistance to a broad class of DNA damaging chemotherapeutic agents using Cancer Cell Line Encyclopedia (CCLE) [21] and The Cancer Therapeutics Response Portal (CTRP) Version 2 [22]. The correlation of genetic alterations such as CNVs and response to standard-of-care agents offers the opportunity to identify potential prognostic and/or predictive biomarkers of therapeutic response that may facilitate the stratification of patients with responder versus non-responder signatures.

### Samples and clinical data

Two hundred six DNA copy number profiles for pediatrics sarcoma were collected from the publicly accessible databases, NCBI - Gene Expression Omnibus (GEO) [https://www.ncbi.nlm.nih.gov/sites/GDSbrowser/] and Therapeutically Applicable Research to Generate Effective Treatments (TARGET) [https://ocg.cancer.gov/programs/target]. These data sets included CNVs from OS (*n* = 117), RMS (*n* = 64), and ESFTs (*n* = 25) (Table 1, Additional file 2). Database of Genomic Variants (DGV) [20] specifically, the hg38 DGV provided comprehensive genomic structure variation of healthy individuals for the sarcoma CNV comparison. CNVs of all sarcoma tumors were tested prior to surgery without prior chemotherapy. The median age at diagnosis was 15 years (range 2–20 years). These sarcomas were intermediate to high grade (93%). The detailed clinical sample annotation is listed in Additional file 2.

**Table 1:** Datasets and their source for healthy and pediatric sarcoma patients

Data Type	Source	Platform	Sample size
Healthy Individuals	DGV (Database of Genomic Variants) [13]	BAC Acgh, FISH, OligoACGH, PCR, Sequencing, SNP array and Digital array	22, 255
Osteosarcoma (OS)	GEO (GSE33383) TARGET	Affymetrix Genome-Wide Human SNP 6.0 Array chips, GPL6801	32 85
Rhabdomyosarcoma (RMS)	GEO (GSE24715)	Affymetrix Mapping 250 K Sty2 SNP chips, GPL3720	7
		Affymetrix Human Mapping 50 K Xba240 SNP chips, GPL2005	57
Ewing Sarcoma (ESFTs)	GEO (GSE8398)	Agilent-013282 Human Genome CGH Microarray 44B, GPL2879	25

CCLE project provides a detailed genetic characterization of a large panel of human cancer cell lines (*n* = 947) which includes 24 cancer types [21]. Copy number profiles of 27-sarcoma cancer cells lines previously obtained by Affymetrix SNP Array 6.0 were collected (see Table 2 for cell lines). The sensitivity of drug responses were quantified using the Area Under the Curve (AUC) for 481 candidate cancer drugs in 27 sarcoma cell lines collected from the Cancer Therapeutics Response Portal (CTRP v2.0) [22] and integrated with copy number alterations from the CCLE.

**Table 2:** Sarcoma cancer cell lines

Tissue	Histology	Cell_line	Tissue	Histology	Cell_line
soft_tissue	Rhabdomyosarcoma	A-204	bone	Ewings_sarcoma	RD-ES
soft_tissue	Rhabdomyosarcoma	Hs 729	bone	Ewings_sarcoma	SK-ES-1
soft_tissue	Rhabdomyosarcoma	KYM-1	bone	Ewings_sarcoma	SK-N-MC
soft_tissue	Rhabdomyosarcoma	RD	bone	Ewings_sarcoma	TC-71
soft_tissue	Rhabdomyosarcoma	RH-30	bone	Osteosarcoma	143B
soft_tissue	Rhabdomyosarcoma	RH-41	bone	Osteosarcoma	G-292
soft_tissue	Rhabdomyosarcoma	SJRH30	bone	Osteosarcoma	HOS
soft_tissue	Rhabdomyosarcoma	TE 441.T	bone	Osteosarcoma	Hs 870.T
soft_tissue	Rhabdomyosarcoma	TE 617.T	bone	Osteosarcoma	Hs 888.T
bone	Ewings_sarcoma	A-673	bone	Osteosarcoma	MG-63
bone	Ewings_sarcoma	CADO-ES1	bone	Osteosarcoma	SJSA-1
bone	Ewings_sarcoma	Hs 822.T	bone	Osteosarcoma	T1–73
bone	Ewings_sarcoma	Hs 863.T	bone	Osteosarcoma	U-2 OS
bone	Ewings_sarcoma	MHH-ES-1			

## Results

### Comparative analysis of CNVs from OS, RMS, and ESFTs to healthy population genomes

**Fig. 1:**
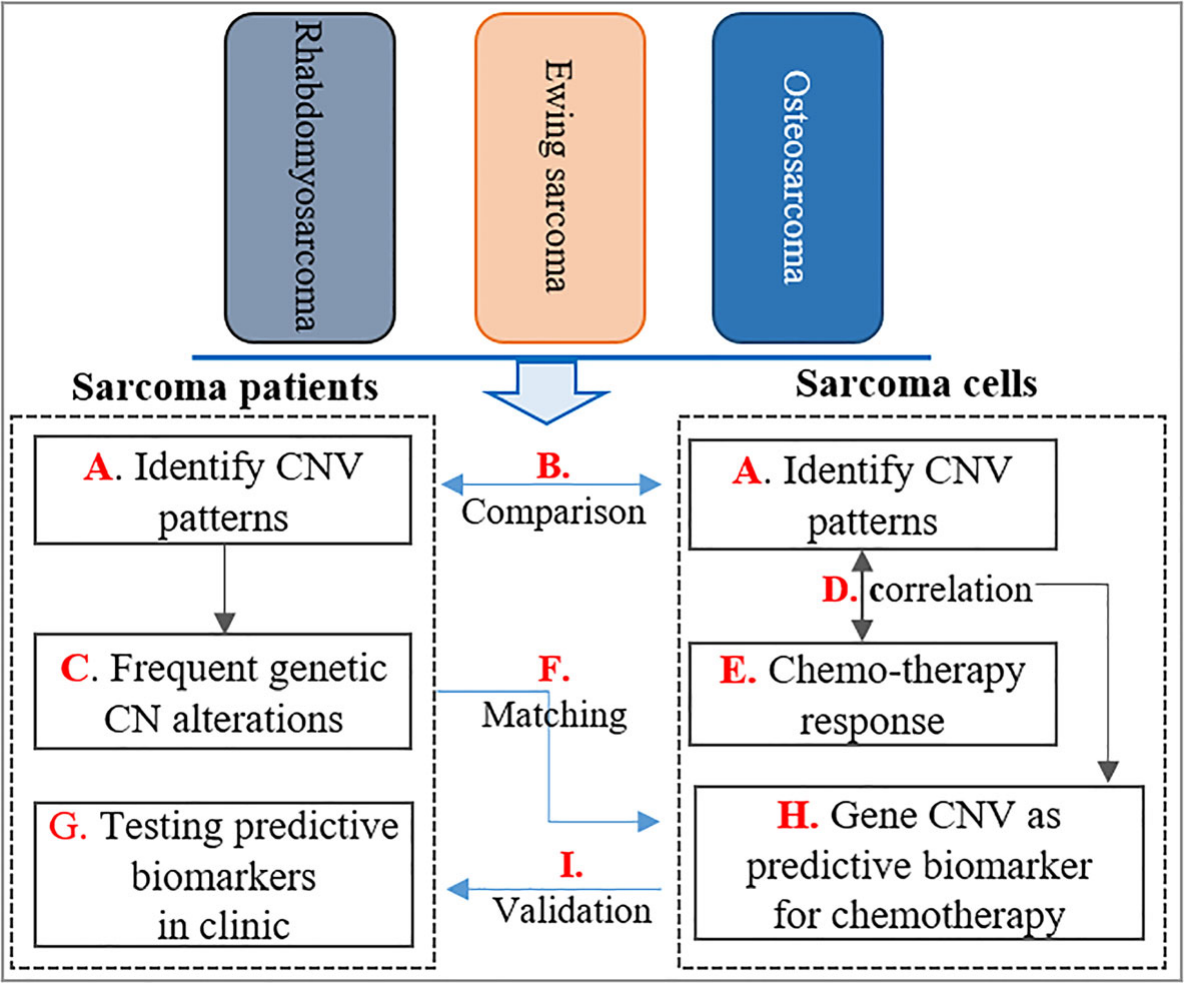
Integration of genomic CNVs to chemotherapy-response for identification of predictive biomarkers of therapeutic response in pediatric sarcoma tumor biopsies and cell lines. **a** CNV patterns were detected in 206 sarcoma (OS, RMS, and ESFTs) patients as well as in 27 sarcoma cell lines (OS, RMS, and ESFTs). **b** The CNVs identified from sarcoma cells were also compared with the profile observed in the 206 sarcoma patients. **c** Frequent CNVs were identified by literature review from PUBMED and compared with 206 patients CNVs. **d**,**e** Large screening to evaluate drug response associated with CNVs using a Pearson Correlation calculation was completed to identify potential predictive biomarkers of therapeutic response in these sarcomas. **f** Matching sarcoma patients CNVs to sarcoma cells. **g**, **h**, **i** Significant predictive biomarkers of sensitivity and resistance to chemotherapy are obtained. Significant predictive biomarkers of sensitivity and resistance to chemotherapy will be identified for further exploration

A comprehensive assessment of CNVs using high-resolution array CGH (Affymetrix SNP) array was completed on OS, RMS and ESFT sarcoma patients (Fig. 1). Genes or regions frequently comprised of these CNVs were identified by comparing whole genome CNVs of a healthy population from DGV 22,255 samples.

**Fig. 2:**
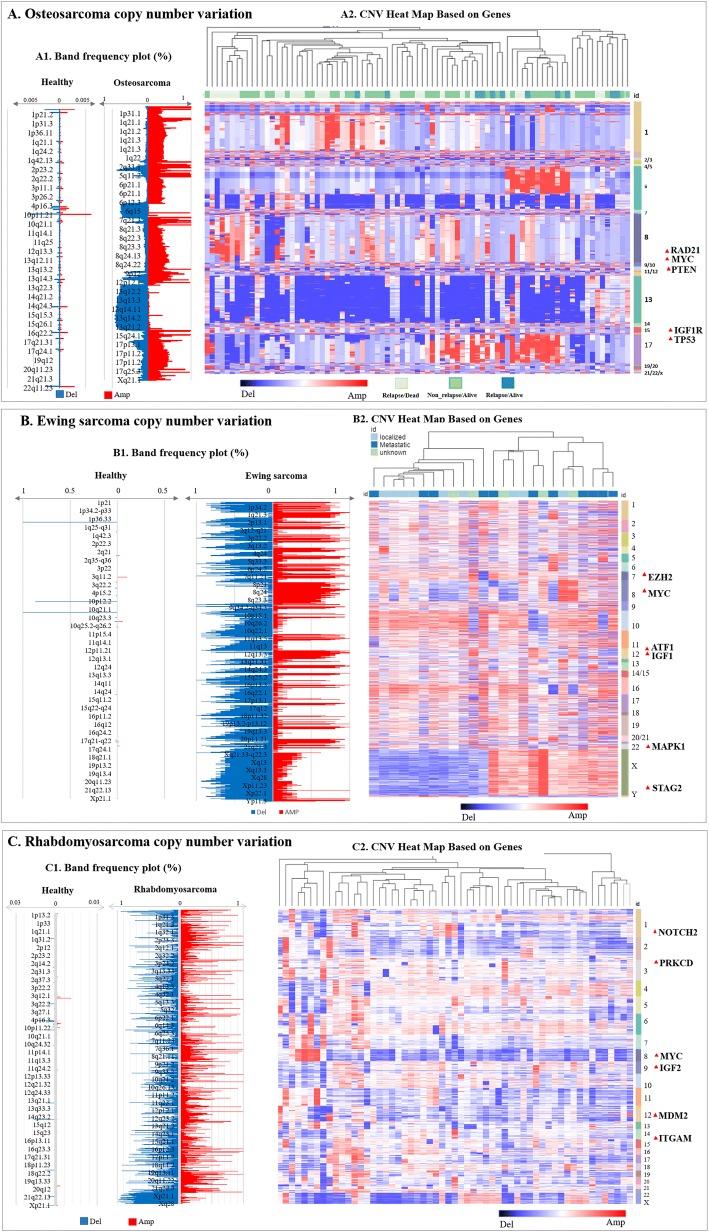
Detection of CNVs by aCGH in 206 patient tumor biopsies from pediatric sarcomas (OS, RMS, and ESFTs). (**a**1) Frequency plots of genome CNV. Band frequencies (CNV deletion and amplification) in OS (horizontal axis, from 0 to 100%) are plotted as a function of chromosome location (from 1pter to the top, to 22qter to the bottom) and compared with the healthy population (DGV). Horizontal lines indicate chromosome boundaries. Positive and negative values indicate frequencies of tumors showing copy number increase and decrease, respectively, with amplifications of copy number (in red) and deletions of copy number (in blue). (**a**2) Unsupervised hierarchical clustering of genome CNVs measured for OS on whole genome with largest gain/lost standard variation. Red indicates increased DNA copy number (CNV gain/amplification) and blue indicates decreased DNA copy number (CNV loss/deletion). Below the dendrogram, each column represents a clinical sample; the status of sample collection is denoted in shades of green (see key at bottom of heat map for Relapse/Dead, Non-relapse/Alive, and Relapse/Alive); each row indicates genes and associated chromosomes. (**b**1) Band frequency plots CNVs (deletion and amplification) among ESFTs compared with healthy individuals. (**b**2) CNVs for ESFT patients were analyzed as described in A2. Below the dendrogram, color at top of each column indicates the diagnosis of the clinical samples (localized disease, metastasis, or non-metastatic). Each row indicates genes and associated chromosome. (**c**1) Band frequency plots of the healthy individuals and RMS patients were analyzed as described in A2. (**c**2) CNVs for RMS patients were analyzed as described in A2

CNV analyses and stratification based on amplification and/or deletion frequencies were conducted for OS, RMS, and ESFTs (Fig. 2a-c). Hierarchy clustering analyses for delineating the pattern of genomic CNVs were conducted to stratify sarcoma patients based on their relapse and metastasis status where CNV distributions greater than the 85% range represented amplification (OS = 2.710, ESFTs = 0.147, RMS = 0.7) and less than the 15% range signified deletion (OS = 1.414, ESFTs = − 0.1467, RMS = − 1.213) (Fig. 2a-c). CNVs that were in between these thresholds for each sarcoma type were considered as having no change in CNVs.

OS (*n* = 117) had the most common gain (copy number amplification) in chromosomes 8, 12, 21, and X*,* while the most common loss (copy number deletion) was found in chromosomes 2, 10, and 13 (Fig. 2 A1). Combination of copy number amplification and deletion were observed in chromosomes 1, 10 and 12 which are comprised of genes amplified or deleted in OS pathogenesis such as RAD21, MYC, PTEN, IGF1R, and TP53 (Fig. 2 A2) [5, 7, 10, 11, 23–26] (details in Additional file 3: Table S1). Frequency analyses of CNV amplifications and deletions in the healthy population indicated the existence of CNVs in regions such as 1q21, 10p11, and 15q25 (Fig. 2 A1).

ESFTs (*n* = 25) also exhibited the presence of copy number gains in chromosomes 1, 8, and 12 (Fig. 2 B1). Deletions (copy number loss) were found in chromosomes 10, 11, and X (Fig. 2 B1)*.* Smaller aberrations were found at chromosome regions: 11q24, 22q12, 5p, 7q, and 9p (Fig. 2 B1). Some genes associated with recurrent ESFT included EZH2, MYC, ATF1, IGF1, MAPK1, FGFR1 and STAG2 (Fig. 2 B2) [2, 4, 6, 9, 27–29].

In RMS (*n* = 64), amplifications (copy number gains) were found in chromosomes 2, 8, 12, and 20. (Fig. 2 C1). Whereas, recurrent loss of heterozygosity (LOH) of chromosomes 1, 7, 14, and X was detected (Fig.2 C1). Genes of interest in RMS that were amplified or deleted and may contribute to the disease pathogenesis/progression included NOTCH2, PRKCD, MYC, IGF2, MDM2 and ITGAM (Fig. 2 C2) [3, 17, 18, 30, 31]. The details are shown in Additional file 3: Table S3.

**Fig. 3:**
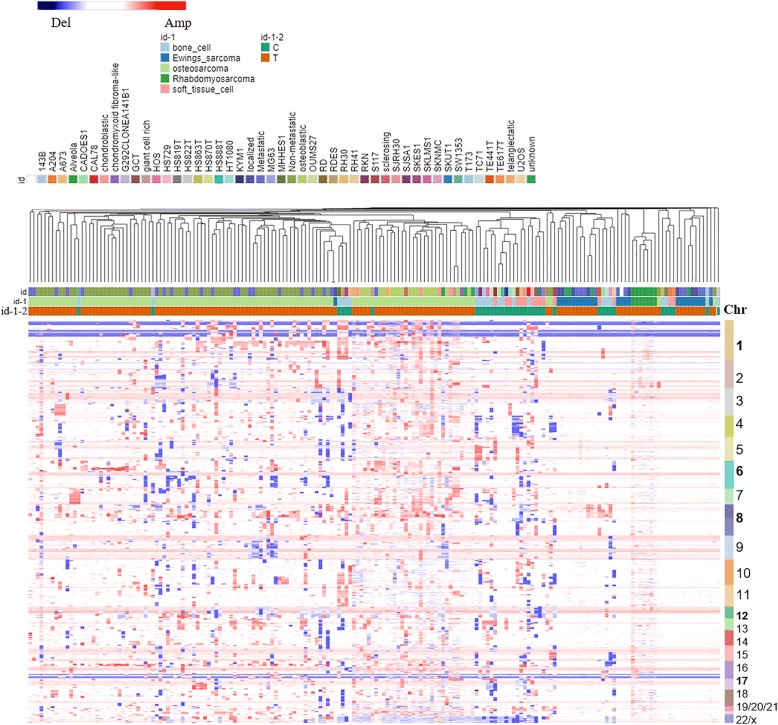
Systematic comparison of CNVs in OS, RMS and ESFT in 206 patient tumor biopsies and 27 sarcoma cell lines. Mark as id = sample name; id-1 = sarcoma type; id-1-2, C = cell line, T = tumor sample. Note: Unknown items in “id” denote sarcoma samples whose exact diagnosis/status at the time of the analysis was not known

**Fig. 4:**
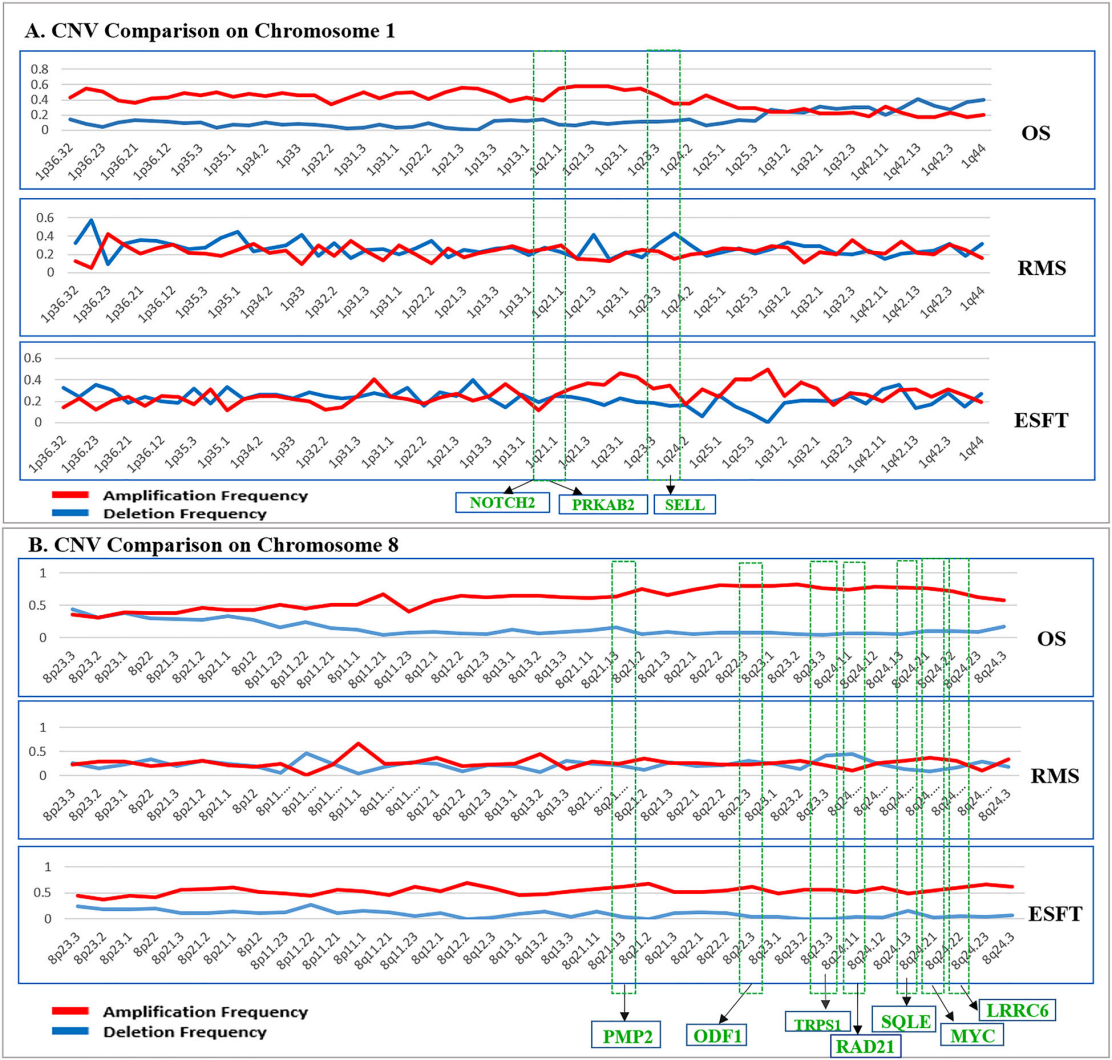
Comparison of CNVs in chromosomes 1 and 8 in OS, RMS, and ESFT patient tumors. **a** CNVs present on chromosome 1 among pediatric sarcomas (OS, RMS, and ESFTs). **b** Variation of amplification and deletion frequencies across different band regions in chromosome 8. Several genes associated with sarcoma progression are highlighted in green. Red denotes the amplification frequency while blue represents the deletion frequency

There are 5417 overlapping genes among OS, ESFTs and RMS at the whole genome level compared to normal healthy controls (Fig. 3). The loss of heterozygosity (LOH) of chromosomes 1, 7, 14, and X was detected in OS, ESFTs and RMS respectively (Additional file 4). A common pattern of copy number gains in chromosome 8 and 12 was found in OS, RMS and ESFT. The specific segment that was amplified in chromosome band 8q23-q24 included MYC, PMP1, ODF1, TRPS1, RAD21, SQLE, FAM49B and LRRC6. Moreover, MYC, which is located in 8q24.21, showed the highest amplification frequency of 0.78 in OS, 0.69 in ESFTs, and was not amplified in RMS with a frequency of 0.32. However, the pattern of CNV exhibited differences among OS, RMS and ES on chromosome 1. The gene SELL had increased copy number amplifications in OS compared to ESFT and RMS. Transcription factor genes on chromosome 1 such as NOTCH2 (deletion), PRKAB2 (amplification) and SELL (amplification) also shared similar copy number alterations in all three types of sarcomas (Figs. 3 and 4).

### Significant CNVs associated with prognostic biomarkers of pediatric sarcomas (OS, RMS, ESFTs)

Based on extensive literature review of all three sarcomas [2–22], [https://www.ncbi.nlm.nih.gov/sites/GDSbrowser/], [23–37], functional outcome of the most frequent CNVs associated with poor outcome was compiled. These gene sets for each sarcoma type is listed in Additional file 3. The top 63 frequently amplified or deleted genes in OS, RMS and ESFTs were previously shown to be associated with recurrent in OS, RMS and ESFTs and are shown in Table 3. All of genes were annotated and mapped to chromosome level by Hg19. See methods section for details on data analysis [38, 39], [http://www.affymetrix.com/support/technical/byproduct.affx?product=500k], [40, 41]. In addition, druggable targets denoted in DrugBank (https://www.drugbank.ca/) are included.

**Table 3:** CNVs (amplifications and/or deletions) frequently found in the 63 genes that serve as prognostic biomarkers for pediatric sarcomas (OS, RMS, and ESFTS)

			OS frequency		RMS frequency		ESFT frequency		Sarcoma types (Yes = 1)		
NO	Band	Gene	Amp	Del	Amp	Del	Amp	Del	OS	RMS	ESFT
1	7p11.2	EGFR^a^	0.250	0.094	0.087	0.109	0.438	0.000	1	1	1
2	12q13.3	GLI1	0.156	0.375	0.891	0.000	0.313	0.188		1	1
3	2q36.1	PAX3	0.063	0.531	0.413	0.000	0.250	0.063		1	1
4	7q36.1	EZH2^a^	0.344	0.094	0.413	0.000	0.313	0.125		1	1
5	8q24.21	MYC	0.781	0.031	0.043	0.326	0.625	0.000	1	1	
6	8q24.22	LRRC6	0.625	0.031	0.391	0.043	0.625	0.063	1	1	
7	8q24.13	MTSS1	0.625	0.063	0.283	0.000	0.438	0.000	1	1	
8	15q11.2	MKRN3	0.219	0.188	0.065	0.109	0.438	0.125	1	1	
9	11q24.2	ST3GAL4	0.156	0.281	0.174	0.130	0.438	0.125	1	1	
10	15q22.2	TPM1	0.094	0.156	0.000	0.261	0.125	0.063	1	1	
11	11q23.1	IL18	0.063	0.375	0.043	0.283	0.000	0.500	1	1	
12	11p15.4	TRIM21	0.063	0.500	0.174	0.109	0.000	0.563	1	1	
13	10q23.31	ACTA2	0.000	0.906	0.087	0.326	0.125	0.375	1	1	
14	8q22.3	ODF1	0.875	0.031	0.413	0.087	0.688	0.188	1		
15	8q24.13	SQLE	0.875	0.031	0.696	0.022	0.938	0.063	1		
16	8q24.11	RAD21	0.781	0.031	0.304	0.022	0.625	0.063	1		
17	8q23.3	TRPS1	0.656	0.063	0.283	0.000	0.500	0.063	1		
18	8q21.13	PMP2	0.594	0.094	0.913	0.000	0.125	0.375	1		
19	8q24.13	TMEM65	0.531	0.063	0.217	0.087	0.500	0.188	1		
20	1q24.2	SELL	0.688	0.063	0.674	0.000	0.188	0.125	1		
21	15q26.3	SNRPA1	0.688	0.125	0.000	1.000	0.125	0.188	1		
22	22q11.21	MAPK1^a^	0.406	0.219	0.000	0.587	0.000	0.688	1		
23	15q26.3	IGF1R	0.313	0.094	0.130	0.065	0.125	0.000	1		
24	15q26.1	KIF7	0.563	0.063	0.065	0.435	0.375	0.000	1		
25	12p13.31	CD163	0.500	0.063	0.174	0.152	0.125	0.125	1		
26	16p13.3	MAPK8IP3	0.000	0.813	0.630	0.043	0.688	0.000	1		
27	10q22.1	NUDT13	0.000	0.781	0.739	0.000	0.125	0.438	1		
28	10q22.1	P4HA1	0.000	0.688	0.935	0.000	0.125	0.313	1		
29	10q23.1	TSPAN14	0.000	0.688	0.043	0.413	0.000	0.750	1		
30	11q13.1	CFL1	0.406	0.125	1.000	0.000	0.063	0.313		1	
31	9q22.33	ALG2	0.625	0.031	1.000	0.000	0.500	0.000		1	
32	1q21.1	PRKAB2	0.406	0.063	1.000	0.000	0.313	0.188		1	
33	16p11.2	ITGAL	0.063	0.375	0.978	0.000	0.375	0.250		1	
34	7q21.2	PEX1	0.438	0.094	0.978	0.022	0.063	0.438		1	
35	3p21.1	PRKCD^a^	0.125	0.156	0.978	0.000	0.063	0.250		1	
36	12q21.1	THAP2	0.344	0.219	0.978	0.000	0.438	0.063		1	
37	19q13.33	AP2A1	0.125	0.063	0.935	0.000	0.063	0.375		1	
38	16p11.2	ITGAM	0.031	0.406	0.913	0.000	0.063	0.250		1	
39	10p14	KIN	0.031	0.563	0.913	0.000	0.125	0.375		1	
41	12q15	MDM2	0.156	0.187	0.391	0.130	0.125	0.500		1	
40	11p15.5	IGF2^a^	0.1875	0.1875	0.283	0.0217	0.75	0		1	
42	11q25	B3GAT1	0.063	0.125	0.000	1.000	0.813	0.000		1	
43	11q13.1	PYGM	0.563	0.063	0.000	1.000	0.313	0.063		1	
44	11q13.1	RELA	0.031	0.688	0.000	1.000	0.625	0.063		1	
45	3p14.1	PSMD6	0.125	0.656	0.000	1.000	0.250	0.000		1	
46	1p12	NOTCH2	0.500	0.000	0.000	1.000	0.375	0.125		1	
47	12p13.2	ETV6	0.156	0.156	0.239	0.000	0.750	0.000			1
48	5q32	PDGFRB^a^	0.063	0.531	0.065	0.870	0.750	0.000			1
49	7p12.3	IGFBP3	0.156	0.188	0.065	0.000	0.688	0.000			1
50	7p21.2	ETV1	0.156	0.063	0.152	0.000	0.563	0.000			1
51	7q33	CREB3L2	0.281	0.094	0.087	0.152	0.125	0.000			1
52	17q21.31	ETV4	0.094	0.250	0.196	0.196	0.563	0.063			1
53	10p11.21	ANKRD30A	0.094	0.438	0.196	0.000	0.500	0.125			1
54	4q12	KIT^a^	0.219	0.063	0.000	0.130	0.375	0.000			1
55	12q23.2	IGF1^a^	0.125	0.094	0.130	0.043	0.250	0.000			1
56	21q22.2	ERG	0.469	0.063	0.109	0.022	0.188	0.063			1
57	20q13.2	NFATC2	0.313	0.219	0.000	0.326	0.188	0.063			1
58	11q24.3	FLI1	0.156	0.125	0.043	0.348	0.188	0.125			1
59	xq25	STAG2	0.031	0.813	0.326	0.022	0.125	0.750			1
60	xp11.4	BCOR	0.000	0.938	0.130	0.217	0.438	0.563			1
61	17q12	TAF15	0.188	0.313	0.370	0.022	0.125	0.500			1
62	6p22.3	KIAA0319	0.375	0.031	0.022	0.391	0.063	0.500			1
63	12q13.12	ATF1	0.281	0.125	0.000	0.326	0.313	0.438			1

Note: ^a^denotes gene with druggable targets by DrugBank annotation. Designation of “1” indicates there is literature to support that specific gene is deleted or amplified for that sarcoma type. All frequency calculation is based on 206 sarcoma patients CNVs

### Comparison of CNVs between patient sarcoma tumors and sarcoma cell lines

**Fig. 5:**
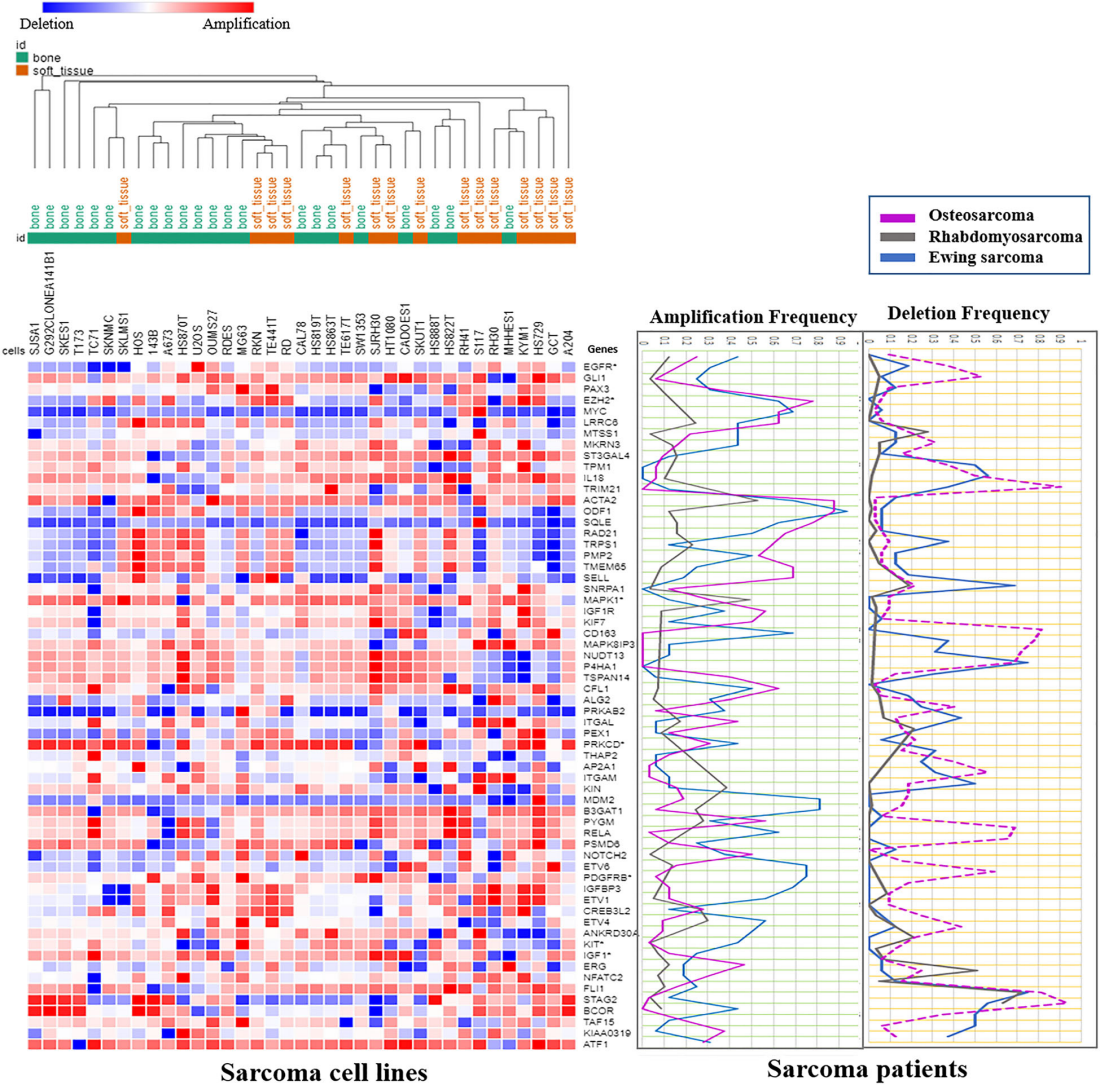
Comparison of CNVs between sarcoma patients and sarcoma cell lines. The left panel represents the CNVs on 27 sarcoma cells, where green is type of bone sarcoma and brown is type of soft tissue sarcoma. The right panel represents the amplification or the deletion frequency of copy number variation in sarcoma patients, which is associated with CNVs of the sarcoma cells

As described above, 63 genes that serve as potential prognostic biomarkers were extracted from bone and soft tissue sarcoma cell lines described in the CCLE where CNVs were categorized based on their high frequencies of amplifications and deletions (Table 2). A hierarchy clustering was used to identify cluster patterns across bone and soft tissue sarcoma cell lines (Fig. 5, left panel). Associated CNV amplification frequencies in sarcoma patient samples were compared with sarcoma cell lines (Fig. 5, right panel). EGFR amplification was showed to be amplified in 25% of the patient samples and in 24% of the sarcoma cell lines. However, MYC was amplified in 60% of the OS and ESFT patient samples while but it was only amplified in 8% of the sarcoma cell lines. IGF1R was amplified more frequencely in OS patients 31.3% than in RMS 13% and ESFT 12.5%.

### Linking CNV profiles in pediatric sarcoma samples to drug sensitivity in sarcoma cell lines with similar CNV profiles

**Fig. 6:**
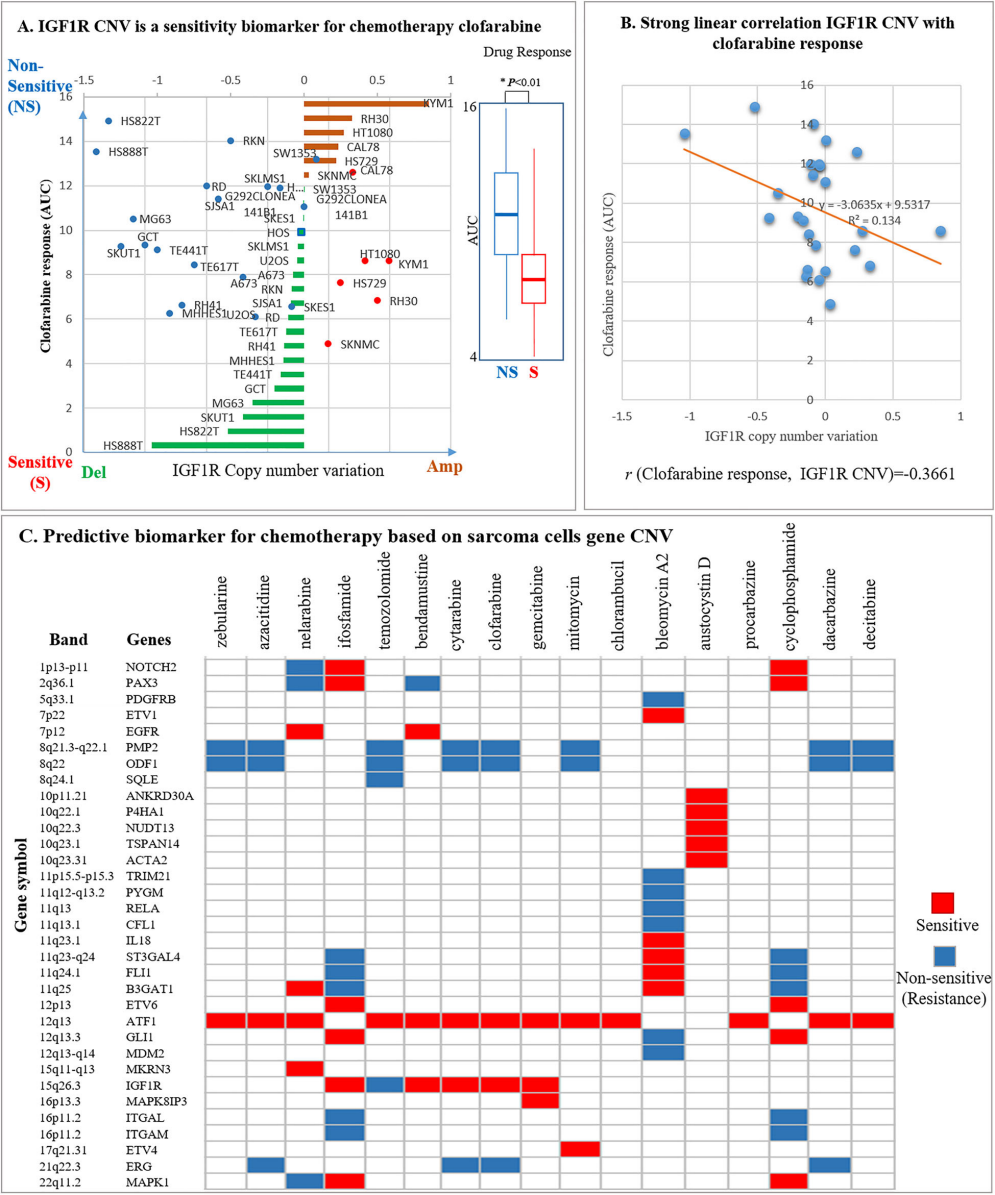
CNVs as predictive biomarker for chemotherapy in pediatric sarcoma. Gene IGF1R and clofarabine response provide an example of data integration. IGF1R gene amplification correlates with increasing sensitivity to clofarabine in pediatric sarcoma. **a** Correlation of IGF1R CNV and relative sensitivity to clofarabine. The IGF1R CNV status (deletion or amplification) for each cell line is presented in the middle of the panel (Del = gene deletion denoted in green; Amp = gene amplification denoted in red). The *y* axis signifies the response of each cell line to clofarabine and is presented as the area under the curve (AUC) to drug response. Blue circles = cell lines with IGF1R deletion; Red circles = cell lines with IGF1R amplification (left panel); Drug response data compiled as non-sensitive (NS) versus sensitive (S) cell lines,*p < 0.01, IGF1R deletion vs. IGF1R amplification, right panel **b** Strong linear correlation between IGF1R CNV and clofarabine response. Blue circles = cellular response to clofarabine; y-axis = AUC and x-axis = CNV of IGF1R. (**c**) CNVs as chemotherapy biomarkers in sarcoma. The sensitive and non-sensitive biomarker selection is based on the threshold where a threshold of *p* < 0.05 and correlation coefficient *r* > 0.35 denotes non-sensitivity and *r* < − 0.35 signifies sensitivity

To elucidate if the CNVs identified in pediatric sarcomas, it could be used to guide selection of therapies that will improve clinical outcome. We next investigated the extent of drug sensitvity in the sarcoma cell lines based on CNVs (CCLE, Table 2). Drug sensitivity correlated with amplifications and/or deletions frequently found in the pool of 63 genes harboring CNVs (Table 2). The database CTRP provides drug-response data of sarcoma cells. Therefore, evaluation and comparison of drug-response with the identified CNVs was integrated from the cell line CCLE and the drug response CTRP databases. Pearson Correlation analysis between CCLE and CTRP indicated that 33 CNVs from 27 sarcoma cell lines had a positive and/or negative correlation with drug response to 17 DNA damaging agents (Fig. 6). For example, IGF1R copy number amplification correlated with sensitivity to clofarabine (Fig. 6a, see left panel), and therefore, may serve as a “sensitive” biomarker of therapeutic response to clofarabine. Since lower concentrations of drug were needed to inhibit growth of the sarcoma cell lines with IGFR1 copy number amplications [see right panel that compares AUC of clofarabine in cell lines with IGF1R gene deletion (clofarabine nonsensitive) or amplification (clofarabine sensitive)]. The significant correlation between clofarabine response associated with CNVs in 27 sarcoma cell lines is illustrated in Fig. 6b. Overall, a number of therapeutic predictive biomarkers were found (Fig. 6c). Integration of 33 CNVs with drug response data in sarcoma cell lines uncovered differential sensitivities to commonly used chemotherapeutic drugs.

## Discussion

Pediatric sarcomas encompass a rare group of heterogeneous neoplasms that arise in bone and soft tissues in the body [1]. Despite the multi-modality approach for treating pediatric sarcomas, clinical outcomes for these patients still remains relatively poor due to onset of relapse/recurrence initiated by various molecular alterations [8–10]. While certain pediatric sarcomas like RMS and ESFTs are more genetically defined by having chromosomal translocations, other pediatric sarcomas such as OS are considered to be more genetically complex in nature [23–26]. For instance, ESFTs are genetically characterized by specific chromosomal translocations t(11;22) (q24;q12) in 85% of ESFTs [28]. However, the remaining 15% of ESFTs have other chromosomal translocations, which involve other members of the FET and ETS family [9]. Similarly, alveolar rhabdomyosarcoma is characterized by a chromosomal translocation t(2;13) (q35;q14) or t(1;13)(p36;q14) fusing the PAX3 or PAX7 with FOXO1 [17, 18]. On the contrary, in sporadic osteosarcoma there are various genetic alterations such as aberrations on chromosomes 15q and 8p where inconsistent rearrangements and copy number alteration have been observed [35–37].

Regardless of their genetic landscape, efforts by several multi-institutional groups have been on-going to investigate novel therapeutic options for improving overall survival for these pediatric malignancies. However, even with these advancements, the 5-year survival rates for relapsed/recurrent pediatric sarcoma patients still remain less than 30% [1, 2]. Therefore, along with identifying downstream targets of these molecularly-characterized and complex pediatric sarcomas, it is equally imperative to assess and identify other acquired genetic changes such as CNVs involving genetic amplifications and/or deletions that may provide novel therapeutic options to improve clinical outcomes [29]. Notably, OS, RMS, and ESFT exhibit various CNVs that can serve as prognostic biomarkers for these pediatric sarcomas [23–31]. Our objective for this study was to identify CNVs common to all three of the pediatric sarcomas (OS, RMS, ESFTs) and evaluate the role of these CNVs in response to DNA damaging agents to determine if they are predictive biomarkers of therapeutic response. This comprehensive study investigated band and gene alterations of somatic copy number amplification and deletion in 27 bone and soft tissue sarcoma using aCGH arrays (Affymetrix). Due to increased availability of publicly available datasets, improved and efficient resources for integrative genomic sequencing, and molecular characterization of patient-specific tumors it is now feasible and could be potentially used to guide selection of personalized therapies.

Through our comparative genomic analyses of OS, RMS, and ESFTs and healthy subjects, we identified CNVs (amplifications and deletions) in various chromosomal regions (Fig. 2). Bioinformatics analyses was also conducted to identify the pattern of genomic instability in these pediatric sarcomas. To the best of our knowledge, this is the first study to compare genomic instabilities between OS, RMS, ESFTs and healthy population controls. Genes associated with survival and/ recurrence of these sarcomas with statistical significance were found on long arm of chromosome 8 with much higher amplification frequency observed in OS (0.8–0.92). These include MYC (8q24.21), LRRC6 (8q24.22), MTSS1 (8q24.13), ODF1 (8q22.3), SQLE (8q24.13), RAD21 (8q24.11), TRPS1 (8q23.2), PMP2 (8q21.13), TMEM65 (8424.13). In ESFTs, there is higher amplification frequency (0.5–0.7) for majority of the bands and lower deletion frequency (0–0.1) in chromosome 8. Similar results are obtained in RMS. CNVs, in particular, amplifications involving chromosome 8 have also been reported by other groups in OS, RMS, and ESFTs, thus, further validating our data [23–31]. While further exploration is needed to assess the role and function of many of the amplified genes present on chromosome 8 in pediatric sarcomas, one key gene that has been highly studied in these pediatric sarcomas is MYC, which has a role in various other cancers [36, 37]. MYC is a transcription factor that is known to regulate critical biological functions such as cell cycle, apoptosis, and metabolism [36]. Genetic alterations that result in changes to MYC, such as MYC amplification, can dysregulate its normal function and alter the balance between being a tumor suppressor versus being tumorigenic [36]. Along with chromosomal changes observed in chromosome 8, smaller aberrations in OS, RMS, and ESFTs were also identified at chromosomes 1q, 12q and x. The long arm (1q) of chromosome 1 also signifies amplification with gene SELL showing higher significance in OS. The majority of the bands in the long arm (1q) of chromosome 1 have an amplification frequency 0.2–0.4 while the deletion frequency is between 0.1–0.2 in ES. Several CNV analyses [17, 28, 30, 31] have validated and verified the accuracy of our results.

However, CNVs associated with recurrence in these pediatric sarcomas correlate with poor prognosis by specific chromosomal translocations or variations in OS, RMS, and ESFTs that can serve as prognostic biomarkers for these diseases [4–7]. To date, the correlation between these prognostic biomarkers and their response to therapies still requires further exploration using in vivo pediatric sarcoma models.

We identified CNVs in 63 genes among the three pediatric sarcomas (OS, RMS, and ESFTs) that correlated with the recurrence of the diseases, suggesting CNVs in the 63 genes may provide prognostic biomarkers for these sarcomas. The 63 genes have high frequency of amplifications as well as deletions in these sarcomas. For example, genes such as KIF7, IGF1R and SNRPA1 on 15q16.1-15q16.4 are amplified in OS. In RMS amplification of PAX3 (2q36.1) with frequency of 0.413 was observed, whereas, a high deletion frequency of 0.9–1 was evident in CFL1, ALG2, PRKAB2, ITGAL, PEX1, PRKCD, AP2A1, KIN, ITGAM, THAP2 genes. ESFTs exhibit frequently mutated STAG2 on chromosome Xq25 [2, 40] with a high deletion frequency of 0.75 in our study.

By integrating large-scale drug screening to evaluate drug response profiles of the CNVs identified in 63 genes from 27 sarcoma cell lines it was identified that 33 genes with CNVs had either sensitive or non-sensitive responses to 17 chemotherapies. The CNVs in these 33 genes could serve as potential predictive biomarkers of therapeutic response which still needs to be further explored. An example of this included the CNVs identified in IGFR1 (Table 3). IGFR1 is receptor for the growth hormone insulin growth factor (IGF) which can mediate cell proliferation [26]. Binding of IGF to IGFR1 initiates downstream singling cascades to increase cell proliferation and decrease apoptosis, which is observed in these pediatric sarcomas [26]. Figure 6a, b show that CNVs in IGFR1 result in IGFR1 serving as a sensitive biomarker of therapeutic response to Clofarabine. Clofarabine is a purine nucleoside analog that can inhibit DNA/RNA polymerases and promotes apoptosis of cancer cells [41, 42]. This study provides novel insights into how genetic alterations such as CNVs can potentially serve as both prognostic biomarkers and predictive biomarkers of therapeutic response in pediatric sarcomas. The systems pharmacology approach described here provides a platform to personalize therapies that have could improve clinical outcomes in aggressive pediatric malignancies [43, 44].

## Conclusions

In our study, we evaluated CNVs as well as their frequencies of amplification (copy number gain) and deletion (copy number loss) in a large cohort of OS, ESFTs, and RMS patient samples and sarcoma cell lines. To the best of our knowledge, this is the first study screening genomic-profiling (CNVs) of aggressive pediatric sarcomas and assessing their drug-responses to potentially improve therapeutic and clinical outcomes in these aggressive diseases. Our future studies will be focused on functionally validating identified targets using in vivo modeling approaches and evaluating their roles as a potential predictive and/or prognostic biomarker in our quest to improve the currently dismal therapeutic outcomes in pediatric sarcoma patients.

## Methods

### Data collection

#### Healthy subjects

The comprehensive genomic structure variation data for the healthy individuals was collected from the Database of Genomic Variants [20]. Fifty-five published studies were included in DGV, from the well-known archival SV databases including, dbVar (NCBI) and DGVa (EBI). The latest dataset GRCh 37 (hg19) version released on May 15, 2016 is collected [45]. A total of 488,630 variant records in 22,255 samples were used to study the CNVs representing a total of 14,316 non-redundant individuals across ~ 44 different populations representing both males and females almost equally. Each of these entities contain multiple studies from different analysis. Insertion, deletion, duplication, tandem duplication, novel sequence insertion and mobile element insertion in chromosomes were investigated. All genomic variants in DGV were detected by different experiment methods, including Bacterial Artificial Chromosome (BAC) and oligonucleotide-based chromosomal Comparative Genomic Hybridization (Oligo-cCGH), aCGH, fluorescence in situ hybridization (FISH), polymerase chain reaction (PCR), sequencing, single nucleotide polymorphism (SNP) array and Digital array. The latest data consists of 44% from microarray studies, 33% from sequencing and 3% from FISH/PCR and Optimal Mapping. The size of the DNA segment for CNV ranges from 50 bp to 3 Mb, with lesser number of variants in the range of 50 bp to 1 Kb range. This is because the majority of the CNV detected using microarray is large-scale CNV. All genome region segments of CNVs were obtained and mapped to genes and bands for further study.

#### OS

One hundred seventeen OS are collected from TARGET and GEO database respectively, where both data sets were tested by Affymetrix Genome-Wide Human SNP 6.0 Array chips (GEO platform accession ID, GPL6801). 85 samples from TARGET [12] were obtained and segments of CNVs with level 3 data were selected. 32 CEL files of CNV profile were obtained from GEO accession ID, GSE33383 with high-grade OSs. Both datasets provide clinical information about each subject including recruitment, demographics, survival and physical examinations (Table 4).

**Table 4:** Demographics and clinical characteristics of sarcoma patients

Sarcoma Type	Data Source		Clinical Information	Number of Samples
OS	TARGET	Stage1	Relapse	41
			Non-Relapse	44
		Stage2	Metastatic	22
			Non-Metastatic	63
		Gender	Female	37
			Male	48
		Vital Status	Alive	55
			Dead	30
		Age	At the time of Diagnosis (days): 1299–11,828	85
High Grade OS	GEO-GSE33383	Age	At the time of Diagnosis (days): 3072–14,965	32
ESFTs	GEO-GSE8398	Gender	Female	11
			Male	9
			Unknown	5
		Stage	Metastatic	11
			Localized	9
			Unknown	5
RMS	GEO-GSE24715	Histology Subtype	ARMS (Alveolar rhabdomyosarcoma)	64

#### ESFTs

CGH profiling of 25 ESFT tumor samples, from GEO accession ID, GSE8398 (65), were scanned on Agilent-013282 Human Genome CGH Microarray 44B (GEO platform accession ID, GPL2879). All 25 sample CEL files were used for data analysis. The datasets provided detailed clinical information of samples, such as disease stage, site of disease, occurrence of metastasis and patient status (Table 4).

#### RMS

CGH profilings of 64 alveolar RMS genome variation, from GEO accession ID, GSE24715. 7 sample genome variation was tested by Affymetrix Mapping 250 K Sty2 SNP chips (GPL3720 in GEO, 238378 probe sets), while 57 samples were tested on Affymetrix Human Mapping 50 K Xba240 SNP Array (GEO platform ID: GPL2005). The raw CEL files were used to generate the CNVs, which were further analyzed for deletion and amplification frequencies.

#### Cancer cell line encyclopedia

All segments of copy number variations for 27 sarcoma cell lines were collected from CCLE, which were tested by Affymetrix Genome-Wide Human SNP Array 6.0. Log 2 transformed segment values were used for further analysis.

### Data pre-processing and gene annotation

All CEL files obtained from GEO were quantified using the Bioconductor package in R. The Oligo library was used to obtain the copy number values for the DNA segments by MAS 5.0 algorithm. The normalized *log*_2_ ratio (healthy/tumor) on probe-sets was annotated to genes for further analysis. All continuous variable CNV will be changed into five level value, where − 2 is significant deletion, − 1 is deletion, 0 is no change of CNV, 1 is amplification and 2 is significant amplification. Base on whole CNV histogram distribution of a particular array platform, the CNV value is larger than top 5% range, we set CNV as 2; when the CNV is large than 15% range and less than top 5% range, it sets as 1. The CNV value is less than negative 5% range; we set CNV as − 2. The CNV value is less than negative 15% range and larger than negative 5% range, it sets − 1, others sets 0.

The HGNC (HUGO Gene Nomenclature Committee, http://www.genenames.org/) database provides researchers with standard gene names for the human genome to avoid the complexity of multiple overlapping and conflicting nomenclature systems. The database currently consists of around 24,000 genes and their corresponding approved gene symbols. Each gene has a unique HGNC ID which makes it easier to identify the gene type. Genes were also annotated with other information including gene synonyms, uniprot ids, refseq ids, previous gene symbols and a functional description about each gene, all of which aids in integrating the information from the NCBI or other databases [46].

By software Bedtools ‘intersectBed’, we mapped genome region segments of CNV to gene symbols by GRCh37/hg19 genome annotation file [38]. All segment data records were changed into individual genes and associated bands on chromosomes. For this work, all the genes were mapped to their standard HGNC name using the annotations from Ensemble-Biomart [45] for multi-data integration and comparison.

### High-resolution array comparative genomic hybridization (aCGH) Chip and Assay

1. Affymetrix Genome-Wide Human SNP 6.0 Array (GPL6801) contains 934,946 SNPs and 946,371 non-polymorphic probes for the detection of CNVs. Enzymes Nsp I and Sty I were used in parallel in the assay to digest and fragment DNA. Probes on the SNP Array 6.0 are targeting sequences that may sit on fragments cut by either enzymes or both. All SNP probes occur in a Nsp, Sty or Nsp + Sty fragment, but the CN probes occur only Nsp and Nsp + Sty fragments (not Sty-alone fragments). The total genomic DNA (500 ng) was digested with Nsp I and Sty I restriction enzymes into fragments and ligated to adaptors that recognize the cohesive 4 bp overhangs. A generic primer that recognizes the adaptor sequence was used to amplify adaptor-ligated DNA fragments. The amplified DNA is then labeled and hybridized to a SNP Array 6.0. PCR conditions will be optimized to preferentially amplify fragments in the 200 to 1100 bp size range. The Birdsuite software is applied here to identify rare CNVs from the Affymetrix SNP 6.0 array via a one-dimensional Gaussian mixture model (GMM) [39]. We matched 946,371 CNV probes to 22,891 genes by GEO released platform GPL6801 annotation.
2. The GeneChip® Human Mapping 500 K Array is one of the aCGH chips designed by Affymetrix Company. It is comprised of two arrays, each capable of genotyping on average 250,000 SNPs (approximately 262,000 for Nsp arrays corresponding to CNV and 238,000 for Sty arrays associated with SNPs. GPL3720 in GEO is a platform of Affymetrix Mapping 250 K Sty2 SNP Array, which is a subset of the GeneChip® Human Mapping 500 K Array Set. The array has probes for CNVs and each marker can be interrogated with up to five probes, ensuring cross-verification for data integrity [http://www.affymetrix.com/support/technical/byproduct.affx?product=500k]. Affymetrix Human Mapping 50 K Xba240 SNP Array is the GeneChip® Mapping 100 K Set for SNPs (GPL2005). It is comprised of a set of two arrays that enable genotyping of greater than 100,000 SNPs with a single primer. All CEL files are normalized to digital number by Affymatrix NET ‘cdf’ package in R [http://www.affymetrix.com/support/technical/byproduct.affx?product=500k].
3. Agilent-013282 Human Genome CGH Microarray 44B (GPL2879) is a high performance 60-mer oligonucleotide, allowing genome-wide survey and molecular profiling of DNA copy number changes on a single chip. It consists of 44,290 60-mer oligonucleotide probes, 7321 genes, empirically validated in multiple model systems, spanning coding and noncoding sequences with average spatial resolution of 35 *kb*.

### Copy number amplification and deletion frequency calculation based on gene or band

Copy number alterations were derived from aCGH chips and measured using log2 ratios of the fluorescence intensities from two channels (Cy3 and Cy5), one for the target sample and the other for the reference sample. For a given gene (or region), a negative log2 ratio is an indication of a loss, and a positive log2 ratio is an indication of a gain. If the log2 ratio equals zero, the target sample and the reference sample have the same copy number for that given gene (or region). However, it should be noted that different platforms can demonstrate differences in amplifications and deletions even when using the same strategy to normalize data from CEL file to digital number. To compensate for this variability in platforms, we used equal quartiles for integration of all pertinent datasets.

The threshold for amplification and deletion decisions were based on the quantile distribution for any gene set, the extreme values present in lower threshold (less than 15% range, such as <= − 0.146768) and in upper threshold (more than 85% range, such as > = 0.147642) are considered as significant threshold for deletion and amplification respectively. Similarly, for the band, the extreme values present in lower quartile (less than 15% range, <= − 0.16109) and in upper quartile (more than 85% range, > = 1.7007) are considered as significant for deletion and amplification respectively.

For each of the genes, the total number of samples studied, total number of observed gains in those set of samples and total number of observed loss in the same set of samples is calculated. The amplification fraction and deletion fraction for each of the genes is then calculated using the formula below:

(i). Deletion frequency for each gene = Total number of observed losses for a specific gene/Total number of samples for that gene.
(ii). Amplification frequency for each gene = Total number of observed gains for a specific gene/Total number of samples for that gene.

To study the amplification and deletion fraction for a unique band similar steps are applied as for these genes. The total number of genes is calculated in each band to a given chromosome. The amplification and deletion fraction for these bands is calculated using the formula below:

(i) Deletion frequency for each band = Total number of observed losses for a band/Total number of samples for a specific gene × Total number of genes for that specific band.
(ii) Amplification frequency for a band = Total number of observed gains for a band/Total number of samples for a specific gene × Total number of genes for that specific band.

### Large scale of drug screening

Cancer Therapeutics Response Portal (CTRP v2.0) provides more than 481 small molecules screening on 664 cancer cell lines [22]. Twenty-seven sarcoma cancer cell lines were included from CTRP. Pharmacologic area under the dose-response curve test AUC is used to describe the drug response reaction. Drug efficacy estimation of AUC values, a nonparametric spline regression technique with the constraint that each drug’s higher dose concentration provides at least equal or higher drug efficacy (inhibition) than its lower concentration was applied for estimating the drug activities across each drug’s experimental range of dose concentration. The successive parabolic interpolation for one-dimensional optimization, implemented with the *nlminb* routine of R, was used to obtain the final AUC estimates by inverting the dose-effect curves.

### Statistical analysis

All code and programming were done using R. Pearson correlation coefficient is used to calculate association between drug response AUC and copy number variation in each of gene. GENE-E software (https://software.broadinstitute.org/GENE-E/) is used for clustering analysis and visualization.

## Additional files

Additional file 1: All NCCN biomarkers for sarcoma,including diagonosis and therapy determination (XLSX 17 kb)

Additional file 2: Samples annotation for sarcoma patients and cell lines in the study. (XLSX 45 kb)

Additional file 3: Comprehensive literature reviews were also conducted to collect gene CNV amplification and deletion that can be predictive of clinical outcome in three types of sarcoma from the PubMed repository. (XLSX 122 kb)

Additional file 4: Integrated CNVs from sarcoma cells and sarcoma patients of OS, RMS and ESFT. (XLSX 3277 kb)

## Abbreviations

aCGH: array-based CGH
AUC: Area under the dose-response curve
BAC: Bacterial artificial chromosome
CCLE: Cancer cell line encyclopedia
CGH: Comparative genomic hybridization
CNV: Copy number variation
CTRP: Cancer therapeutics response portal
DGV: Database of genomic variants
ESFTs: Ewing’s sarcoma family of tumors
FISH: Fluorescence in situ hybridization
GEO: Gene expression omnibus
HGNC: HUGO gene nomenclature committee
HUGO: Human genome organisation
LOH: The loss of heterozygosity
NCCN: National comprehensive cancer network
OligoCCGH: Oligonucleotide-based Chromosomal CGH
OS: Osteosarcoma
PCR: Polymerase chain reaction
RMS: Rhabdomyosarcoma
SNP: Single nucleotide polymorphism
TARGET: Therapeutically applicable research to generate effective treatments
